# Novel eco-friendly [1,2,4]triazolo[3,4-*a*]isoquinoline chalcone derivatives efficiency against fungal deterioration of ancient Egyptian mummy cartonnage, Egypt

**DOI:** 10.1007/s00203-022-03395-7

**Published:** 2023-01-07

**Authors:** Neveen S. Geweely, Mona M. Soliman, Rania A. Ali, Hamdi M. Hassaneen, Ismail A. Abdelhamid

**Affiliations:** 1 Department of Botany and Microbiology, Faculty of Science, Cairo University, Giza, 12613 Egypt; 2 Department of Mummies and Human Remains Conservation, Central Department of Conservation and Restoration, Project Sector, Ministry of Tourism and Antiquities, Cairo, Egypt; 3 Department of Chemistry, Faculty of Science, Cairo University, Giza, 12613 Egypt

**Keywords:** Fungal deterioration, triazolo[3,4-*a*]isoquinoline chalcones, Archeological objects, Cartonnage, Minimum inhibitory concentration, Mummy, Microorganism

## Abstract

Fungal deterioration is one of the major factors that significantly contribute to mummy cartonnage damage. Isolation and molecular identification of thirteen fungal species contributing to the deterioration of ancient Egyptian mummy cartonnage located in El-Lahun regions, Fayoum government, Egypt was performed. The most dominant deteriorated fungal species are *Aspergillus flavus* (25.70%), *Aspergillus terreus* (16.76%), followed by *A. niger* (13.97%). A newly synthesized series of tetrahydro-[1,2,4]triazolo[3,4-a]isoquinoline chalcone derivatives were synthesized and evaluated for their antifungal activities in vitro against the isolated deteriorated fungal species (*Aspergillus flavus, A. niger, A. terreus*, *Athelia bombacina*, *Aureobasidium iranianum*, *Byssochlamys spectabilis*, *Cladosporium cladosporioides*, *C. ramotenellum*, *Penicillium crustosum*, *P. polonicum*, *Talaromyces atroroseus*, *T. minioluteus* and *T. purpureogenus*). The most efficient chalcone derivatives are new chalcone derivative numbers **9** with minimum inhibitory concentration (MIC) ranging from 1 to 3 mg/mL followed by chalcone derivatives number **5** with MIC ranging from 1 to 4 mg/mL.

## Introduction

The scientific study of ancient human mummified skeletal remains has gained much attention over the past years with the improvement and progression of laboratory techniques (DeAraujo et al. 2016). The cartonnage in ancient Egypt appeared as an alternative from the deceased features through which the spirit can reach the body again if the mummy was damaged (Ali et al. 2016). Several factors lead to the deterioration of mummies including environmental conditions, physical damage, biological damage (Abdel-Maksoud and El-Amin 2013). Microorganisms (bacteria, actinomycetes, and fungi) cause the most serious damage to mummies (Naji et al. 2014). Ancient human remains and mummies were damaged by microbial species in processes that liquefying, degrading, or mineralizing different materials causes corrosion, fouling, rotting, declining, and disfiguring (DeAraujo et al. 2016).

Many fungal and bacterial species require available moisture for their development on the object surface (Valentin 2001). A temperature range for the growth of microorganisms is 30 °C (Valentin 2002), which controls the reaction, particularly collagen, which is the main component of mummy skin (Maekawa 1998), High temperatures can prevent the microbial growth but can cause the mummified skin to stiffen and become more susceptible to cracking and chemical breakdown. Increasing the relative humidity (RH) of museum air was believed to be the main factor responsible for microbial growth (Zaitseva 2010)**.** A high relative humidity (RH) that arrives at 65℅ or higher aids in the decomposition of mummies (Valentin 2002). pH value aid in the growth of fungi and bacteria, whereas the fungi prefer acidic environments pH 6 is suitable for growth (Abdel-Maksoud and El-Amin 2013).

The deteriorating fungi can colonize books, clothing, manuscripts, mummies, and paintings causing cracking, crushing, discoloration, spots, and loss of material strength by enzymatic degradation, the production of metabolites, and mechanical attacks (Mansour et al. 2020). The fungal metabolites that accelerate the deterioration of the different archaeological substrata are carbon dioxide, water, organic acids (acetic, citric, oxalic acids, and others), and inorganic acids, specific pigments (melanin black pigments, and other colored pigments), volatile organic compounds (alcohols, aldehydes, and ketones), nitrogen-containing compounds (amino-acids, and beta-glucans), lipids and polyols (glycerol, and others), enzymes (cellulase, protease, amylase, and gelatinase), and secondary metabolites (mycotoxins and antibiotics) (Farooq et al. 2002; Borrego and Perdomo 2012; Lavin et al. 2016; Kakakhel et al. 2019).

Xerophilic and halophilic fungi share in mummy degradation due to xerophiles can grow on dry meat, and halophilic fungi can also tolerate high salinity. Xerophiles fungi (*Cladosporium cladosporioides*, *Penicillium chrysogenum*, and *Aspergillus flavus*) can produce mycotoxin that may also affect human health by breathing the high concentration of aflatoxins and fungal spore when opening the burial chamber after thousand years of being closed (Mansour 2018). Xerophilic fungi including *Aspergillus penicillioides*, *Eurotium repens*, *E. rubrum*, *Penicillium roqueforti*, and *Wallemia sebi* grow faster under acidic conditions (Gock et al. 2003). During the post-excavation storage and repair phases, waterlogged archaeological objects is at a significant risk of biological destruction (Geweely 2022).

Fungal species require necessary nutrients (nitrogen, vitamins, and minerals) for growth, which can obtain it from the meat of mummies (a major source of fats, protein, and vitamins) (Jay et al. 2008; Baltic and Boskovic 2015).

Exposure to fungal spores, metabolites, mycotoxins, and result in critical negative health impacts on the skin and respiratory system of library workers, conservators, and visitors (Sterflinger and Pinzari 2012). So, it is crucial to inhibit microbial proliferation to preserve archaeological objects and human health. Several physical and chemical strategies have been employed for heritage conservation including ultraviolet, laser, gamma rays, and traditional chemical compounds. These methods have various limitations including temporary actions, high cost, and toxic effects (Michaelsen et al. 2013; Sequeira et al. 2017). Various mechanical methods are incapable of preventing the growth of microorganisms completely. Many microorganisms have become resistant to the applied conventional antifungal agents (Kakakhel et al. 2019). To overcome these limits, the development of new and more efficient antimicrobial agents is required for application in materials preservation (Campana et al. 2020).

There is an urgent need to develop an alternative green eco-friendly strategy achieving high efficiency, cost-effectiveness, long-term effect, and safety for the preservation of historical artifacts against microbial colonization (Fouda et al. 2019). The isolation and screening of natural products are an important part of biocide discovery, where several successful drugs and molecules have been discovered via this route (Menezes and Diederich 2019). Novel inhibition of some pathogenic fungal and bacterial species by new synthetic phytochemical coumarin derivatives (Geweely 2009a). The development of new and more efficient antimicrobial agents for application in monument preservation is required to inhibit or prevent fungal growth on archaeological artifacts (Campana et al. 2020).

Chalcones (1,3-diaryl-2-propen-1-ones) are bio-precursors of flavonoids and isoflavonoids in plants (de Mello et al. 2018). Nowakowska (Nowakowska 2007) stated that chalcone forms the backbone of many natural products and is widely distributed as the main component in vegetables, fruits, teas, and other plants with a great interest for their biological activities. The biological profile of chalcone attracted the attention of organic chemists worldwide to design and develop new chalcone derivatives (Rani et al. 2019). Chalcones exhibited a variety of biological activities including antibacterial (Nanjundaswamy et al. 2022), antifungal (Lahtchev et al. 2008), anticancer (Mohamed et al. 2012, 2014, 2018, 2022; Tantawy et al. 2019; Fathi et al. 2021; Srilaxmi et al. 2021; Helmy et al. 2021; WalyEldeen et al. 2022, 2023; Kamel et al. 2022; Sroor et al. 2022) anti-inflammatory (Rojas et al. 2003), antimalarial (Larsen et al. 2005), and antiviral (Cheenpracha et al. 2006). The mechanism of action for chalcones can be act via the α,β-unsaturated carbonyl moiety as Michael acceptor, thioredoxin reductase, and inhibition of microtubule formation (Menezes and Diederich 2019).

These can contribute to a better understanding and developing new flavonoid-based antifungal agents as multi-target agents in the treatment of fungal deterioration. The different bioactivities and simple structures of natural chalcones have attracted the attention of many researchers, and many structural alterations have been conducted to improve activities (Wang et al. 2016; Jin 2019).

The aim of the present work is conservation of the tested ancient Egyptian mummy cartonnage from microbial deterioration using novel chalcone derivatives as eco-friendly, efficient, safe, and new alternative preservatives for cultural heritage. In our study, eight [1,2,4]triazolo[3,4-*a*] isoquinoline chalcone derivatives were targeted against thirteen fungal species isolated from the tested ancient Egyptian mummy cartonnage located in El-Lahun regions, Fayoum government, Egypt.

## Materials and methods

### Source of isolation

A deteriorated ancient Egyptian mummy cartonnage from the Supreme Council of Antiquities (SCA) excavation in El-Lahun regions, Fayoum government, Egypt was tested. The ancient Egyptian mummy cartonnage belonged to the late period of ancient Egypt and its number of the registry is 236. The cartonnage composed of multilayers of fabric structure (linen bandages and papyrus wrappings), which are covered by a gesso layer and colored with different pigments.

**Fig. 1:**
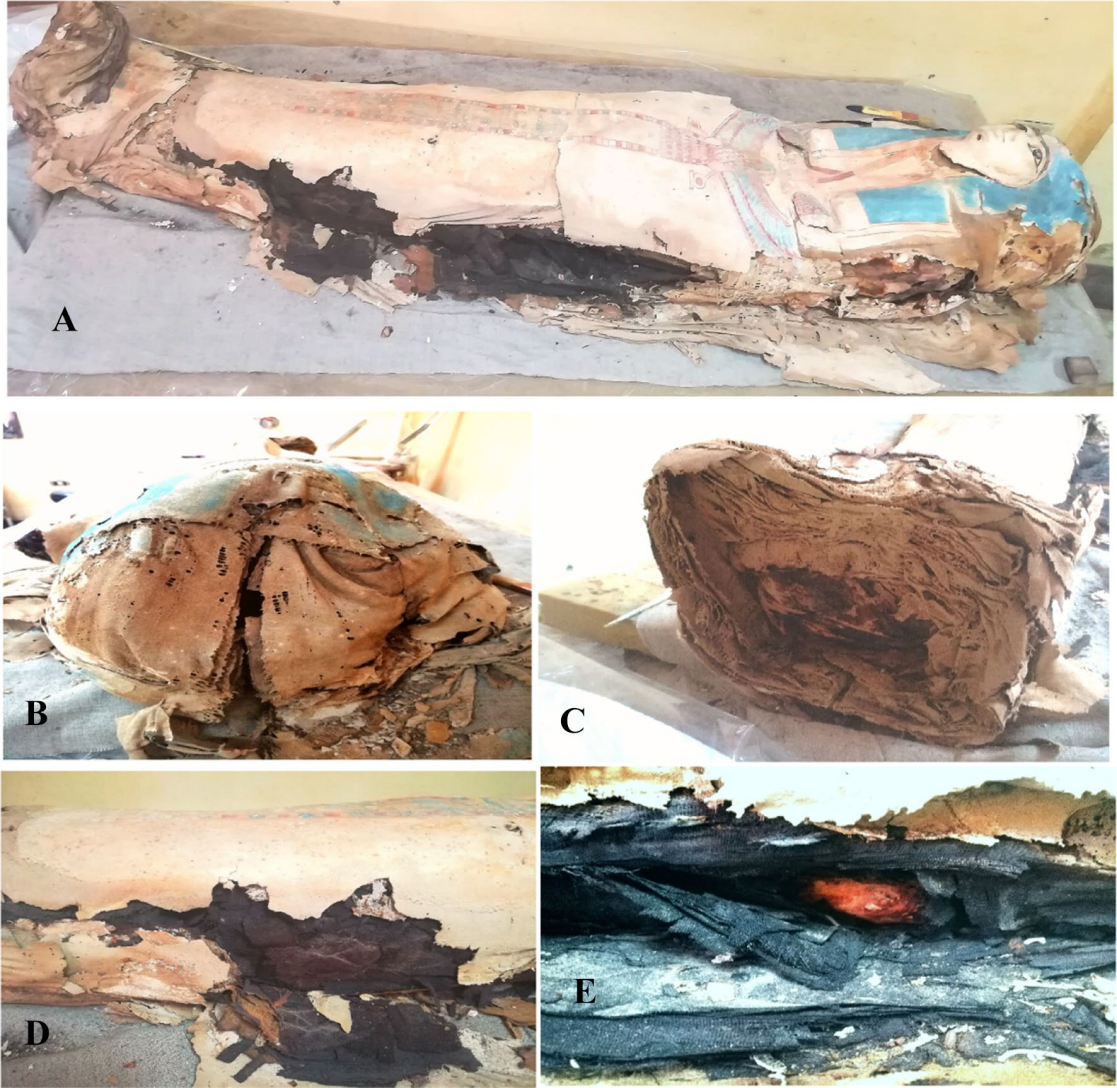
The tested ancient Egyptian mummy cartonnage belonged to the Supreme Council of Antiquities (SCA) excavation in El-Lahun regions, Fayoum, Egypt. **A** The whole ancient Egyptian mummy cartonnage with damage a long two sides of cartonnage with downfall of gesso layer, **B** Appearance of small holes and degradation of linen bandage with longitudinal cracks, **C** Missing parts in lower area of feet accompanied with loss in structure cohesion of fabric layer, **D** Decay of mummy tissues with roasting of linen bandages, and **E** Dryness and gaps in linen bandages with change in its color to dark brown and black resulting in appearance of bone

The tested ancient Egyptian mummy cartonnage was found in a bad state with many deterioration aspects Fig. 1. It suffered from loss in painting layer accompanied with the occurrence of damage a long two sides of cartonnage, and downfall of gesso layer. Fabric structure suffered from severe fragile and damaged parts with change in its color tending toward dark brown or black, while some linen parts were roasted. The presence of longitudinal cracks in the head and feet area was also observed. The mummy tissues were decayed which result in the appearance of bones.

### Isolation and identification of deteriorated fungal species from the tested ancient Egyptian mummy cartonnage

The fungal species were isolated from the twelve different deteriorated parts (top of the vertex, face mask, neck, chest area, abdomen area, left and right sides of cartonnage, leg, the lower part of feet, bones, mummy tissues, and roasted linen) of the tested ancient Egyptian mummy cartonnage by swabbing with sterile cotton swabs. In the laboratory, Potato dextrose agar dishes were streaked with the inoculated swabs. The plates were incubated at 27 °C for 7 days (Naji et al. 2014). The isolated fungi were identified microscopically (Samson et al. 1981; Moubasher 1993)**.**

### Molecular identification of the isolated fungal species from the tested ancient Egyptian mummy cartonnage

Molecular identification was performed for the fungal isolates from tested ancient Egyptian mummy cartonnage. DNA extraction was performed by Quick-DNA™ Fungal Microprep Kit (Zymo Research) according to manufacturers’ protocol. The fungal sample (2 mg) was mixed with 95 μL water, 95 μL solid tissue buffer (blue) and 10 μL proteinase K and incubated at 55 °C for 2 h. Then, the mixture was centrifuged at 12,000×*g* for one minute. The aqueous supernatant has been transferred to a clean tube with the addition of 600 μL genomic binding buffer and completely mixed. It was transferred in the collecting tube of the Zymo-Spin™ IIC-XL column and centrifuged at 12,000×*g* for 1 min. A DNA pre-wash buffer (400 μL) was added in a new collection tube of the Zymo-Spin™ IIC-XL column and centrifuged at 12,000×*g* for 1 min. The genomic DNA wash buffer (700 μL) was added to the Zymo-Spin™ IIC-XL column followed by centrifugation at 12,000 × g for 1 min and the flow-through was discarded. The genomic DNA wash buffer (200 μL) was again added and centrifuged at 12,000×*g* for 1 min, then the collection tube was discarded. The Zymo-Spin™ IIC-XL column was transferred to a sterilized Eppendorf tube and the elution buffer was added directly to the column matrix, and then incubated for five minutes, followed by centrifugation at 12,000×*g* for 1 min to elute the DNA. The ultra-purified DNA was stored at − 20 °C (Phoku et al. 2014).

The polymerase chain reaction (PCR) was carried out in a 50 μl final volume using MyTaq™ Red Mix (Sigma). According to manufacturers’ protocol, the PCR reactions set up included 25 μL of MyTaq™ Red Mix, 8 μL DNA template, 15 μL Nuclease Free Water, 1 μL of 20 μM ITS1 forward primer, and 1 μL of 20 μM ITS4 primer. The two primers: ITS1 forward primer (5′-TCC GTA GGT GAA CCT GCG G-3′) and ITS4 reverse primer (5′-TCC TCC GCT TAT TGA TAT GC- 3′) (Wirya et al. 2020). Amplification of the PCR products was performed using the thermal cycling conditions: initial denaturing cycle for 6 min at 94 °C (1 cycle), 35 cycles of denaturation for 45 s at 94 °C, annealing for 45 s at 56 °C, and elongation for one minute at 72 °C with a final extension cycle for 5 min at 72 °C.

PCR amplified products were evaluated by electrophoresis with low melting 2% TBE agarose gel (2 g agarose in 98 ml Tris–Borate- EDTA buffer). Ethidium bromide was added to the solution and mixed thoroughly. Electrophoresis was done for 15 min at 70 Volt. The electrophoresed DNA was visualized by UV light (Phoku et al. 2014).

Sequencing was made to purified PCR product on GATC Company by the mean of ABI 3730 XL DNA sequencer using forward and reverse primers only by combining the traditional Sanger technology with the new 454 technology. BLAST program available at NCBI Gen Bank databases (National Center for Biotechnology Information) was used to align DNA strand forward and reverse sequence. Isolate identification was performed based on the analysis of hits from mega-blast (highly similar sequences) output (Bhore et al. 2010).

### Chemistry

Melting points were determined on a Stuart melting point device and they are uncorrected. The ^1^H and ^13^C-NMR spectra were recorded in DMSO-*d*_*6*_ as solvent at 300 MHz and 75 MHz, respectively on Varian Gemini NMR spectrometer using Tetramethylsilane (TMS) as internal standard. Chemical shifts are reported in δ units (ppm). The IR spectra were recorded as KBr using a Bruker-vector 22 spectrophotometer FTIR. Mass spectra were measured on a Shimadzu GMSS -QP-1000 EX mass spectrometer at 70 eV. The elemental analyses were performed at the Microanalytical Center, Cairo University.

### Synthesis of tetrahydro-[1,2,4]triazolo[3,4-*a*]isoquinolin-3-yl)-3-arylprop-2-en-1-one (3–10)

A mixture of 3-acetly[1,2,4]triazolo[3,4-*a*]isoquinolines **1a,b** (0.351 g, 1 mmol) and the appropriate arylaldehydes **2a–e** (1 mmol) was dissolved in 20 mL ethanol. Potassium hydroxide (20%, 5 mL) was added to this mixture at 0–5 °C. The reaction mixture was stirred at room temperature for 3 h, then poured over ice containing HCl (1 mL). The mixture was filtered to obtain the solid, washed with water, and dried. The crude product was crystallized from ethanol to afford the chalcone derivatives **3**–**10**.
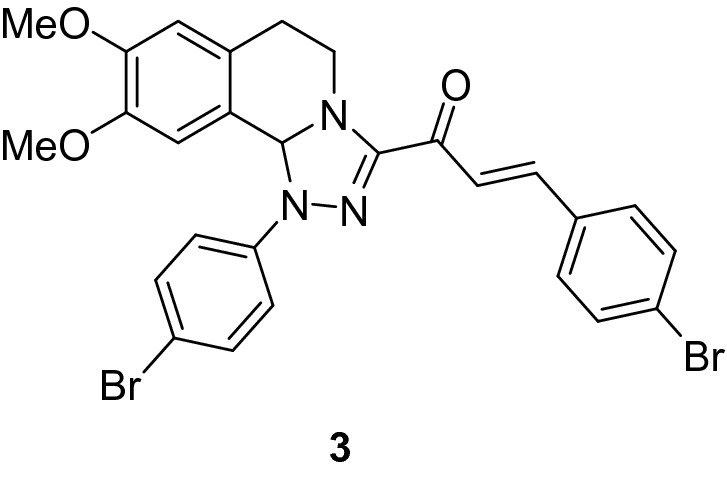


### (*E*)-3-(4-Bromophenyl)-1-(1-(4-bromophenyl)-8,9-dimethoxy-1,5,6,10b-tetrahydro-[1,2,4]triazolo[3,4-*a*]isoquinolin-3-yl)prop-2-en-1-one (3)

Yield: (74%) as an orange solid (from ethanol); m.p 78–80 °C. IR (KBr, cm^−1^): 1656 (CO); ^1^H NMR (300 MHz, DMSO-*d*_*6*_): δ, ppm: 2.74 -2.82 (m, 2H, H6), 3.44 (s, 3H, OMe), 3.47–3.48 (m, 1H, H5), 3.72 (s, 3H, OMe), 3.80–4.11 (m, 1H, H5), 6.61 (s, 1H, H10b), 6.80 (s, 1H, H7), 6.92 (s, 1H, H10), 6.97–7.72 (m, 10H, Ar–H + vinyl-H); MS (EI): m/z = 597 (M^+^). Anal. Calcd. for C_27_H_23_Br_2_N_3_O_3_ (597.31): C, 54.29; H, 3.88; N, 7.04. Found: C, 54.43; H, 3.72; N, 7.19.
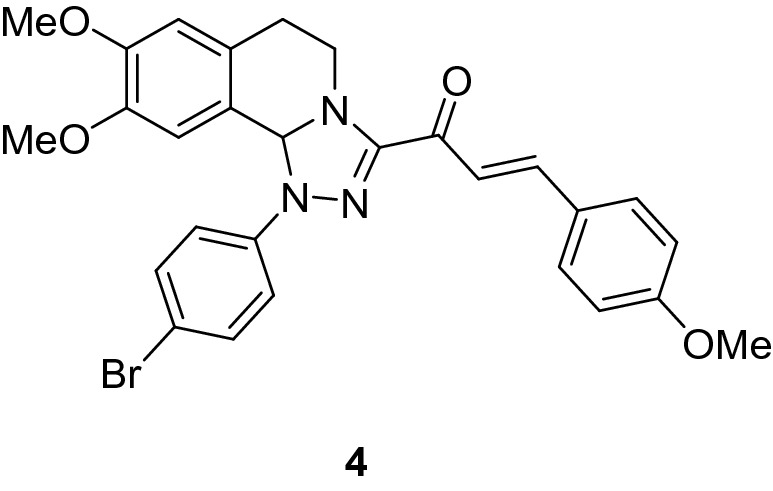


### (*E*)-1-(1-(4-Bromophenyl)-8,9-dimethoxy-1,5,6,10b-tetrahydro-[1,2,4]triazolo[3,4-*a*]isoquinolin-3-yl)-3-(4-methoxyphenyl)prop-2-en-1-one (4)

Yield: (78%) as a yellow solid (from ethanol); m.p 144–146 °C. IR (KBr, cm^−1^): 1653 (CO); ^1^H NMR (300 MHz, DMSO-*d*_*6*_): δ, ppm: 2.58–2.72 (m, 2H, H6), 3.41 (s, 3H, OMe), 3.45–3.55 (m, 4H, H5 + OMe), 3.72 (s, 3H, OMe), 4.22–4.33 (m, 1H, H5), 6.61 (s, 1H, H10b), 6.80 (s, 1H, H7), 6.87 -7.95 (m, 11H, H10 + Ar–H + vinyl-H). MS (EI): m/z = 548 (M^+^). Anal. Calcd. for C_28_H_26_BrN_3_O_4_ (548.44): C, 61.32; H, 4.78; N, 7.66. Found: C, 61.51; H, 4.82; N, 7.50.
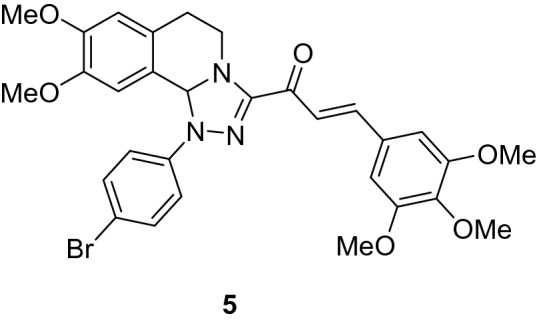


### (*E*)-1-(1-(4-Bromophenyl)-8,9-dimethoxy-1,5,6,10b-tetrahydro-[1,2,4]triazolo[3,4-*a*]isoquinolin-3-yl)-3-(3,4,5-trimethoxyphenyl)prop-2-en-1-one (5)

Yield: (76%) as an orange solid (from ethanol); m.p 170–172 °C. IR (KBr, cm^−1^): 1655 (CO); ^1^H NMR (300 MHz, DMSO-*d*_*6*_): δ, ppm: 2.62–2.81 (m, 2H, H6), 3.48 (s, 3H, OMe), 3.72–3.74 (m, 7H, 2OMe + H5), 3.84 (s, 6H, 2 OMe), 4.23–4.34 (m, 1H, H5), 6.61 (s, 1H, H10b), 6.80 (s, 1H, H7), 6.87 (s, 1H, H10), 7.10 (s, 2H, Ar–H), 7.33–7.61 (m, 6H, Ar–H + vinyl-H). MS (EI): m/z = 608 (M^+^). Anal. Calcd. for C_30_H_30_BrN_3_O_6_ (608.49): C, 59.22; H, 4.97; N, 6.91. Found: C, 59.33; H, 5.12; N, 7.11.
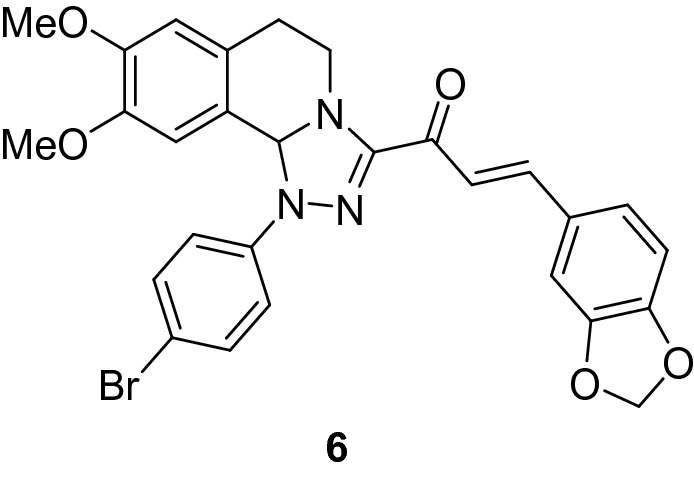


### (*E*)-3-(Benzo[*d*][1,3]dioxol-5-yl)-1-(1-(4-bromophenyl)-8,9-dimethoxy-1,5,6,10b-tetrahydro-[1,2,4]triazolo[3,4-*a*]isoquinolin-3-yl)prop-2-en-1-one (6)

Yield: (80%) as a red solid (from ethanol); m.p 168–170 °C. IR (KBr, cm^−1^): 1655 (CO); ^1^H NMR (300 MHz, DMSO-*d*_*6*_): δ, ppm: 2.64–2.83 (m, 2H, H6), 3.48 (s, 3H, OMe), 3.71–3.72 (m, 4H, OMe + H5), 4.23–4.32 (m, 1H, H5), 6.01 (s, 2H, -OCH_2_-), 6.61 (s, 1H, H10b), 6.79 (s, 1H, H7), 6.87 (s, 1H, H10), 6.96–7.56 (m, 9H, Ar–H + vinyl-H);^13^C NMR (75 MHz, DMSO-*d*_*6*_): δ, ppm: 26.9, 41.4, 55.4, 55.5, 77.2, 101.7, 106.9, 108.6, 111.8, 111.9, 116.2, 120.4, 125.7, 127.1, 128.3, 128.9, 131.9, 142.0, 142.8, 147.1, 148.1, 148.7, 149.7, 149.8, 179.1. MS (EI): m/z = 562 (M^+^). Anal. Calcd. for C_28_H_24_BrN_3_O_5_ (562.42): C, 59.80; H, 4.30; N, 7.47. Found: C, 59.92; H, 4.51; N, 7.62.
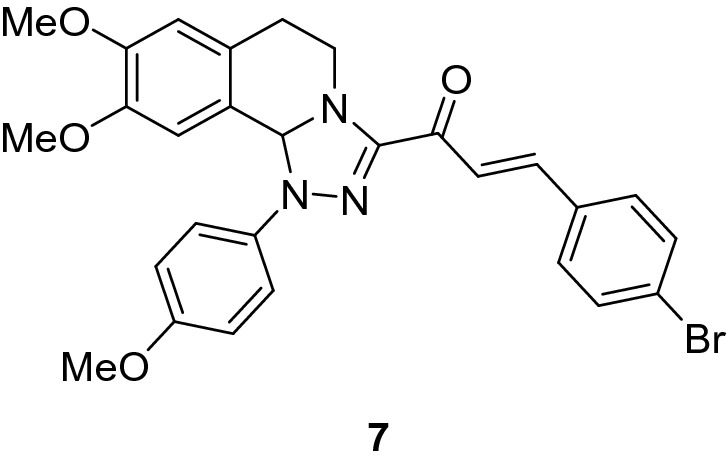


### (*E*)-3-(4-Bromophenyl)-1-(8,9-dimethoxy-1-(4-methoxyphenyl)-1,5,6,10b-tetrahydro-[1,2,4]triazolo[3,4-*a*]isoquinolin-3-yl)prop-2-en-1-one (7)

Yield: (80%) as an orange solid (from ethanol); m.p 142–144 °C. IR (KBr, cm^−1^): 1650 (CO); ^1^H NMR (300 MHz, DMSO-*d*_*6*_): δ, ppm: 2.62–2.78 (m, 2H, H6), 3.39 (s, 3H, OMe), 3.42–3.52 (m, 1H, H5), 3.71 (s, 3H, OMe), 3.75 (s, 3H, OMe), 4.22–4.33 (m, 1H, H5), 6.56 (s, 1H, H10b), 6.73 (s, 1H, H7), 6.93 -7.72 (m, 11H, H10 + Ar–H + vinyl-H). MS (EI): m/z = 548 (M^+^). Anal. Calcd. for C_28_H_26_BrN_3_O_4_ (548.44): C, 61.32; H, 4.78; N, 7.66. Found: C, 61.44; H, 4.89; N, 7.78.
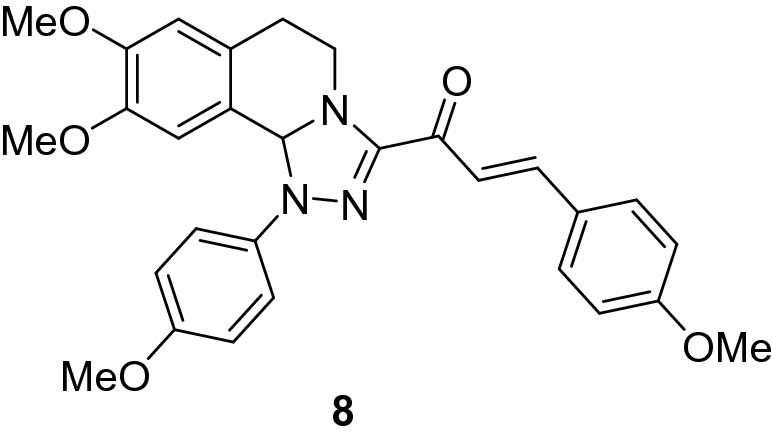


### (*E*)-1-(8,9-Dimethoxy-1-(4-methoxyphenyl)-1,5,6,10b-tetrahydro-[1,2,4]triazolo[3,4-*a*]isoquinolin-3-yl)-3-(4-methoxyphenyl)prop-2-en-1-one (8)

Yield: (79%) as an orange solid (from ethanol); m.p 120–122 °C. IR (KBr, cm^−1^): 1658 (CO); ^1^H NMR (300 MHz, DMSO-*d*_*6*_): δ, ppm: 2.65–2.85 (m, 2H, H6), 3.40 (s, 3H, OMe), 3.43–3.48 (m, 1H, H5), 3.70 (s, 3H, OMe), 3.73 (s, 3H, OMe), 3.75 (s, 3H, OMe), 4.12–4.21 (m, 1H, H5), 6.56 (s, 1H, H10b), 6.73 (s, 1H, H7), 6.82 (s, 1H, H10), 6.95–7.00 (dd, 4H, Ar–H (*J* = 9.3) + 2 vinyl-H (*J* = 15.7 Hz), 7.37 (d, 2H, Ar–H, *J* = 8.7 Hz), 7.54 (d, 2H, Ar–H, *J* = 9.3 Hz), 7.72 (d, 2H, Ar–H, *J* = 8.7 Hz);MS (EI): m/z = 499 (M^+^). Anal. Calcd. for C_29_H_29_N_3_O_5_ (499.57): C, 69.72; H, 5.85; N, 8.41. Found: C, 69.86; H, 5.98; N, 8.65.
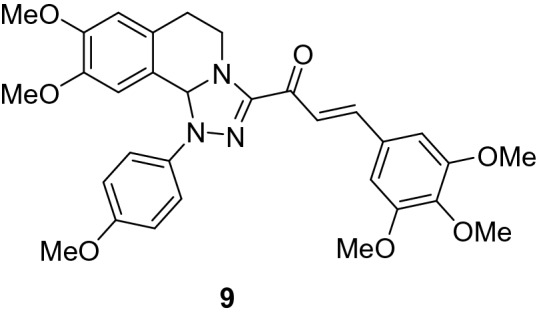


### (*E*)-1-(8,9-Dimethoxy-1-(4-methoxyphenyl)-1,5,6,10b-tetrahydro-[1,2,4]triazolo[3,4-*a*]isoquinolin-3-yl)-3-(3,4,5-trimethoxyphenyl)prop-2-en-1-one (9)

Yield: (74%) as a red solid (from ethanol); m.p 170–172 °C. IR (KBr, cm^−1^): 1656 (CO); ^1^H NMR (300 MHz, DMSO-*d*_*6*_): δ, ppm: 2.64–2.83 (m, 2H, H6), 3.41 (s, 3H, OMe), 3.44–3.46 (m, 1H, H5), 3.70 (s, 3H, OMe), 3.75 (s, 6H, 2 OMe), 3.80 (s, 6H, 2 OMe), 4.24–4.36 (m, 1H, H5), 6.57 (s, 1H, H10b), 6.73 (s, 1H, H7), 6.87 (s, 1H, H10), 6.95–7.72 (m, 8H, Ar–H + vinyl-H). MS (EI): m/z = 559 (M^+^). Anal. Calcd. for C_31_H_33_N_3_O_7_ (559.62): C, 66.53; H, 5.94; N, 7.51. Found: C, 66.72; H, 6.11; N, 7.73.
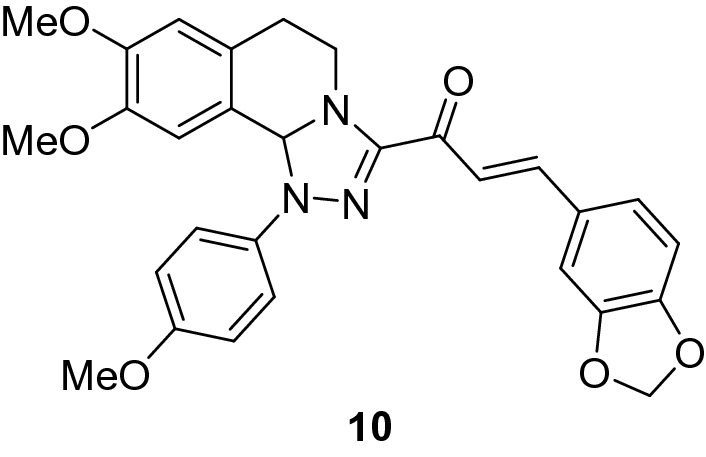


### (*E*)-3-(Benzo[*d*][1,3]dioxol-5-yl)-1-(8,9-dimethoxy-1-(4-methoxyphenyl)-1,5,6,10b-tetrahydro-[1,2,4]triazolo[3,4-*a*]isoquinolin-3-yl)prop-2-en-1-one (10)

Yield: (79%) as an orange solid (from ethanol); m.p 128–130 °C. IR (KBr, cm^−1^): 1658 (CO); ^1^H NMR (300 MHz, DMSO-*d*_*6*_): δ, ppm: 2.61–2.81 (m, 2H, H6), 3.40 (s, 3H, OMe), 3.42–3.52(m, 1H, H5), 3.71 (s, 3H, OMe), 3.74 (s, 3H, OMe), 4.24–4.32 (m, 1H, H5), 6.08 (s, 2H, -OCH_2_-), 6.57 (s, 1H, H10b), 6.73 (s, 1H, H7), 6.88 (s, 1H, H10), 6.95–7.53 (m, 9H, Ar–H + vinyl-H);^13^C NMR (75 MHz, DMSO-*d*_*6*_): δ, ppm: 26.8, 41.5, 55.2, 55.3, 55.5, 78.9, 101.7, 106.9, 108.6, 109.3, 112.0, 114.6, 117.7, 120.8, 125.3, 126.4, 128.6, 129.0, 136.8, 140.9, 146.8, 148.1, 148.5, 149.1, 149.5, 154.5, 178.6. MS (EI): m/z = 513 (M^+^). Anal. Calcd. for C_29_H_27_N_3_O_6_ (513.55): C, 67.83; H, 5.30; N, 8.18. Found: C, 67.94; H, 5.45; N, 8.23.

Effect of the new eight tested chalcone derivatives on the growth of the isolated thirteen deteriorated fungal species from the tested ancient Egyptian mummy cartonnage:-

The antifungal activities of the new eight chalcones (**3**–**10**) were evaluated using agar dilution method (Gupta and Jain 2015). Stock solutions of chalcone compounds were prepared in DMSO. Aliquots of the stock solution were diluted in melted potato dextrose agar at a concentration (1 mg/mL) with vigorously shaking, then poured in Petri plates (9 cm diameter) and let them solidify. Each plate was inoculated at the center with the fungal disc (6 mm) from 7-day-old culture, Itraconazole was used as a standard, and control experiments were performed under similar conditions without the chalcone compounds. All triplicate dishes were incubated at 27 °C for 7 days. Percent of inhibition = (dC − dT) × 100/dC. Where dC is the average diameter of the fungal colony in control and dT is the average diameter of the fungal colony in a treatment group.

### Determination of minimum inhibitory concentration (MIC) for the isolated deteriorated fungal species

The minimum inhibitory concentration (MIC) of the new eight tested chalcone derivatives was determined by the agar dilution method. Stock solutions of synthesized compounds were prepared in DMSO. Aliquots of the stock solution were used to prepare series of subsequent concentrations (1, 2, 3, 4, 5, and 6 mg/mL). The plates were incubated at 27 °C for 7 days. MIC of the new eight tested chalcone derivatives expressed as the lowest concentration of chalcone derivatives (mg of the chalcone derivatives /ml of culture medium) at which no visible growth was occurred compared with control after the incubation time (Gupta and Jain 2015)**.**

## Results and discussion

### Isolation and identification of deteriorated fungal species from the tested ancient Egyptian mummy cartonnage

The data revealed that thirteen fungal species (*Aspergillus flavus, A. niger, A. terreus, Athelia bombacina, Aureobasidium iranianum, Byssochlamys spectabilis, Cladosporium cladosporioides, C. ramotenellum, Penicillium crustosum, P. polonicum, Talaromyces atroroseus, T. minioluteus,* and *T. purpureogenus*) accounting 179 colonies were isolated from the twelve different parts (top of the vertex, face mask, neck, chest area, abdomen area, left and right sides of cartonnage, leg, the lower part of feet, bones, mummy tissues and roasted linen) of the tested ancient Egyptian mummy cartonnage, located in the Supreme Council of Antiquities (SCA) excavation, El-Lahun regions, Fayoum, Egypt (Table 1). The biodeterioration of the tested ancient Egyptian mummy cartonnage might be due to the enzymatic activities of the isolated fungal species as well as their ability to grow and feed on textiles and organic materials (skin and fabrics) as recorded by Kavkler et al. (2015) who indicated that organic materials (protein, acrylic, and calcium organic salts), inorganic materials (calcite, gypsum, and silicate fillers), and various additional materials (the animal glue in the paintings), might have contributed to the fungal growth and acted as additional nutrition sources (Mansour et al. 2020). stated that *Cladosporium cladosporioides, Penicillium chrysogenum,* and *Aspergillus flavus* producing the deterioration of a child's mummy covered by linen wrapping, in Dahshur, Giza, Egypt. Čavka et al. (2010) isolated seven saprophytic genera (*Alternaria spp., Aspergillus fumigatus, Aspergillus nidulans, Chrysosporium spp*., *Monilia spp., Penicillium spp.*, and *Rhizopus spp.*) from different samples (oral, orbital and abdominal cavity and wrapping bandages) of the mummified body from the Archeological Museum in Zagreb, Croatia. Also, Skrlin et al. (2011) isolated *Aspergillus, Bacillus, Penicillium, Sarcina*, and *Shewanella* species from St. Marcian mummy in Rijeka, Croatia. The existence of the isolated thirteen fungal species on the tested ancient Egyptian mummy cartonnage may stay viable for prolonged periods due to that they were more resistant to low levels of water activity (a_w_) as recorded by Teixeira et al. (2018) who stated that the water stress and poor ventilation favor the proliferation of fungal species.

**Table 1:** Total counts and frequency of occurrence of fungal species isolated from the different deteriorated parts of the tested ancient Egyptian mummy cartonnage belonged to the Supreme Council of Antiquities (SCA) excavation in El-Lahun regions, Fayoum, Egypt

Isolation samples/fungal species	Top of vertex	Face mask	Neck	Chest area	Abdomen area	Left side of cartonnage	Right side of cartonnage	Leg	Lower part of foot	Bones	Mummy tissues	Roasted linen	Total isolate	Relative density %	Frequency of occurrence %	
*Aspergillus flavus*	10	–	1	1	11	6	8	1	3	–	1	4	46	25.70	83.33	High
*A. niger*	4	–	2	–	–	3	–	2	1	–	–	13	25	13.97	50.0	Moderate
*A. terreus*	7	3	–	1	2	8	–	–	4	1	3	1	30	16.76	75.0	High
*Aspergilli*	21	3	3	2	13	17	8	3	8	1	4	18	101	56.40	100.0	High
*Athelia bombacina*	–	–	–	–	–	–	–	1	–	–	–	–	1	0.56	8.3	Low
*Athelia *								1					1	0.56	8.3	Low
*Aureobasidium iranianum*	3	–	–	–	–	2	–	–	–	–	1	–	6	3.35	25.0	Low
*Aureobasidium*	3					2					1		6	3.35	25.0	Low
*Byssochlamys spectabilis*	–	–	–	–	–	–	5	–	–	–	–	12	17	9.50	16.7	Low
*Byssochlamys*							5					12	17	9.50	16.7	Low
*Cladosporium cladosporioides*	1	–	–	2	–	–	–	1	1	8	1	–	14	7.82	50.0	Low
*C. ramotenellum*	–	–	1	–	–	–	–	–	–	2	2	–	5	2.79	25.0	Low
*Cladosporium*	1		1	2				1	1	10	3		19	10.61	58.33	Moderate
*Penicillium crustosum*	–	–	2	4	–	6	–	–	–	3	1	–	16	8.94	41.7	Moderate
*P. polonicum*	1	–	–	1	3	–	–	–	1	2	–	–	8	4.50	41.7	Moderate
*Penicilli*	1		2	5	3	6			1	5	1		24	13.41	66.67	High
*Talaromyces atroroseus*	–	2	–	–	–	–	–	–	–	–	–	3	5	2.79	16.6	Low
*T. minioluteus*	–	–	–	–	–	3	–	–	–	–	–	–	3	1.68	8.3	Low
*T. purpureogenus*	–	–	–	–	1	2	–	–	–	–	–	–	3	1.68	16.6	Low
*Talaromyces*		2			1	5						3	11	6.15	33.3	Low
Total count	26	5	6	9	17	30	13	5	10	16	9	33	179	100%		
Number of species	6	2	4	5	4	7	2	4	5	5	6	5	13			

In the present investigation, the highest number of the isolated fungal colonies (33 colonies) was recorded on the roasted linen, followed by the left side of cartonnage (30 colonies), and the linen of the top of the vertex (26 colonies) of the tested ancient Egyptian mummy cartonnage which might be due to the ability of fungal species to feed and grow on cellulosic material by mycelial penetration, and secreting cellulosic enzymes leading to the loss of fiber strength and structure cohesion as recorded by Gutarowska et al. (2017) who recorded that historical cellulosic materials can easily decay by fungal species when exposed to high humidity. Also, Tiano (2002) stated that the most frequent deteriorating fungi on cellulosic textiles with high cellulolytic activities are Ascomycetes and Deuteromycetes Šimonovičová et al. (2015) isolated *Aspergillus fumigatus, A. niger, Coprinellus xanthothrix*, *Penicillium polonicum, P. chrysogenum,* and *Rhizopus stolonifer* from textile samples of mummified human remains in Sládkovičovo, Slovakia. In the Jordanian Museum, the historic textile objects were colonized by *Alternaria alternate,* and *A. tenuissima, Aspergillus flavus, A. fumigatus*, *A. nidulans, A. niger, Penicillium asperum,* and *P. funiculosum,* (Grabek-lejko et al. 2017). The support part of the mummy cartonnage consists mainly of cellulosic textile materials (linen and papyrus), and the binder material that was manufactured from animal or plant glues which make it more susceptible to microbial attack (Abdel-Kareem 2010). Ali et al. (2018) isolated *Aspergillus fumigatus, A. niger, A. tamarii, Cladosporium sp. Fusarium solani,* and *Penicillium chrysogenum* from the cartonnage surface, that consist of painted layer, gesso, and the organic materials (cotton, linen, or wool), which deteriorated by fungal species that result in changes in color, chemical, and physical characteristics of cartonnage. *Alternaria, Aspergillus, Chaetomium, Cladosporium,* and *Penicillium sp.* had been isolated from archaeological cellulosic materials (Fouda et al. 2019).

Five isolated fungal species (*Aspergillus terreus, Cladosporium cladosporioides, C. ramotenellum, Penicillium crustosum,* and *P. polonicum*) accounting 16 colonies were counted from the bone of the tested ancient Egyptian mummy. The obtained result agreed with Šimonovičová et al. (2015) who isolated different fungal species (*Aspergillus candidus, A. terreus, A. venenatus, A. versicolor, A. westerdijkiae, Penicillium chrysogenum,* and *Talaromyces flavus*) from a bone sample of mummified human remains in Sládkovičovo, Slovakia, where *Aspergillus* and *Penicillium* can produce metabolites that able to dissolve organic and inorganic constituents of bone tissue. Jans et al. (2004) stated that fungal colonization is regularly found in archaeological bone in favorable environment conditions (presence of oxygen and moisture) without destruction of the bone microstructure. The fungal metabolites (organic acids) can dissolve bone minerals, followed by collagen hydrolysis by fungal collagenase to harvest nutrients and to use the bone as a medium (Jans 2013). Piñar et al. (2013) found that *Penicillium radicum* was the dominant species in the mummies bone from the Capuchin Catacombs of Palermo, Italy. *Penicillium* species can be involved in the solubilization of the phosphorus that presents in bones minerals as tricalcium phosphate and calcium phosphates.

The tested mummy tissues were deteriorated by six fungal species (*Aspergillus flavus, A. terreus, Aureobasidium iranianum, Cladosporium cladosporioides, C. ramotenellum,* and *Penicillium crustosum*) accounting 9 colonies which may be due to the ability of fungal species to grow on dry meat, which is the major source of protein, fats, minerals, and vitamins in ancient mummies. The obtained results agree with Mansour (2018) who stated that xerophilic and halophilic fungi can help in the degradation of the mummy, due to its ability to grow on dry meat with a high level of salinity at minimum water content in a locked environment. The xerophilic fungus can also grow on leather or fabric bindings (Micheluz et al. 2015).

Arroyo (2009) found *Aspergillus* and *Penicillium* in the ancient proteinous materials. David (2008) mentioned that different fungal species are often seen in ancient tissues as a result of poor storage of the specimen. The proteinaceous objects with a high degree of impurities can increase their susceptibility to fungal attack (Kavkler et al. 2015). Piñar et al. (2013) found *Botryotinia, Giberella, Didymella, Fusarium, Verticillium, Tritirachium, Coprinus,* and *Coniosporium* on the surface and inside the mummy materials from the Capuchin catacombs of Palermo, Italy, while *Phialosimplex* species were the most dominated species on the mummies samples (skin, muscle, and hair), which are keratin- and collagen-rich materials.

The isolated low count of fungal colonies from the face mask of ancient mummy cartonnage, may be due to the presence of heavy metals in the paint layer as recorded by Tiano (2002) who indicated that the presence of heavy metals (chromium, copper, lead, and zinc) in some pigments can increase the resistance of the paint layer against fungal attack. The microbial examination of the tested mummy cartonnage showed that the presence of fungal growth on the paint layer is less frequent than on the fabric layer, which causes serious damage to the mummy. Brittleness and deep cracks were observed as result of fungal damage to archeological oil painting object (Geweely 2006).

In the present investigation, *Aspergilli* were the most dominant deteriorated genera in the twelve samples of the tested ancient Egyptian mummy cartonnage, where 101 colonies were recovered, represented 56.40% of the total fungal colony, and constituted three species (*Aspergillus flavus*, *A. niger*, *A. terreus*)**.** *Aspergilli* and *Penicillia* genera are the most isolated fungal species from human remains, which can adapt well to the conditions offered by a mummified body characterized by low availability of water (Šimonovičová et al. 2015). The obtained data agree with Kraková et al. (2018) who found that *Aspergillus* species were the most widely distributed in the mummy from the castle of Krásna Hôrka, in Slovakia with an abundance of (25–51%), followed by *Penicillium* (3–41%), and *Cladosporium* (14–38%). Also, Naji et al. (2014) noticed that *Aspergilli* was the most common genera (48.94%) isolated from the mummy samples in the Yemen National Museum at Sana’a, where *A. niger* (25.53%) was the most collective species followed by *A. flavus* (10.63%), while *A. fumigatus*, *A. candidus*, and *A. ustus* were isolated in minimal frequency. *Cladosporium* and *Penicillium* were isolated in moderate frequency representing 14.89% and 12.76%, respectively. *Aureobasidium pullulans, Chaetomium thermophilum, Mucor circinelloides, Scopulariopsis koningii, Stachybotrys chartarum, Trichoderma hamatum,* and *Ulocladium chartarum* had been isolated in rare incidence from mummy samples. Lavin et al. (2016) found that *Aspergillus niger*, *Aspergillus flavus*, *Penicillium* sp., *Cladosporium* sp., *Scopulariopsis* sp., *Fusarium* sp. and *Alternaria* sp. produce different pigments which cause esthetic damage.

*Aspergillus flavus* was the most dominant isolated deteriorated fungal species, where the highest count (46 colonies) was recovered from the ten tested parts out of twelve of the tested ancient Egyptian mummy cartonnage accompanied with a high occurrence which constituted 25.70% of the total fungal count. Helmi et al. (2015) isolated *Aspergillus flavus,* and *A. niger* from archaeological funeral masks in Saqqara, Egypt, which caused various deterioration aspects (disintegration, discoloration, cracking, and stains). *A. flavus* can produce a broad range of hydrolytic enzymes which substantial for nutrition and penetration of different organic substrates (Mellon et al. 2007). It can develop also on both complex protein substrates and complex carbohydrate substrates (Teixeira et al. 2018). *Aspergillus terreus* was the second tested species in dominance (30 colonies), followed by *A. niger* (25 colonies) which constituted 16.76% and 13.97% of the total counts from the tested ancient Egyptian mummy, respectively. *A. niger* is a common contaminant on various materials, which produces toxic secondary metabolites (Lee 2004), as well as allergens (Ali et al. 2018)*.* It can be adapted to different environmental conditions by different metabolic mechanisms (Zhang et al. 2017). The degradation action of *A. niger* is performed by the production of pectinases, hemicellulase, xylanase, and arabinase (Pařenicová et al. 2000)**.**

*Aspergillus spp.* was the most frequent species followed by *Penicillium spp.* (24 colonies) and *Cladosporium spp.* (19 colonies). Kavkler et al. (2015) demonstrated that *P. chrysogenum* and *Cladosporium spp.* were the most frequently isolated from cellulosic and proteinaceous materials. *Penicillium* and *Aspergillus* species well-known for their cellulolytic and proteolytic activity on textiles and skin, which menacing the preservation of the mummies. *Penicillium chrysogenum* was isolated from a burial chamber in Upper Egypt. *P. chrysogenum* and *C. cladosporioides* can grow at low temperatures and moisture in an indoor environment. Xerophiles fungi (*Aspergillus flavus Cladosporium cladosporioides,* and *Penicillium chrysogenum*) can produce mycotoxin that may affect human health by breathing the high concentration of aflatoxins and fungal spore when opening the burial chamber after its one thousand years of being closed (Mansour 2018). *Cladosporium cladosporioides* and *C. tenuissimum* were isolated from five historical materials include cellulosic (i.e., cotton, flax, hemp), and proteinaceous (i.e., leather, wool) materials. They cause textile degradation mainly due to their abundant excretion of succinic acid (Kavkler et al. 2015).

Dothideomycetes was the most abundant phylum (29–50%) in (cellulosic material) Library and Archives in Poland, and Czechia. The species of *Cladosporium* (15.827–19.756%), *Aureobasidium* (3.833–16.789%), *Toxicocladosporium* (0.856–3.771%) and *Conidiocarpus* (0–3.255%) were the most common species in this phylum (Krakova et al. 2018). On the other hand, Krakova et al. (2018) detected new species contaminating cultural material includes *Scorias spongiosa, Conidiocarpus caucasicus, Sydowia polyspora, Diaporthe longicolla, Byssochlamys spectabilis,* and *Graphiopsis chlorocephala*, although their presence was in the range of 1–4%. Basidiomycota is not considered as the major fungal contaminant of archival documents, which isolated in lower concentration than Ascomycota species.

*Talaromyces* and *Aureobasidium* species were isolated with a relative density of 6.15% and 3.35% respectively. *Talaromyces helices* (*Penicillium teleomorph*) has been reported from archaeological cellulolytic materials, which uses cellulose as a carbon source to penetrate cracks and migrate between layer and causes detachment (Ali et al. 2014). The least isolated species in order of density was occupied by *Athelia bombacina* which represented 0.56% of the total count. Basidiomycota is not considered the major fungal contaminant of archival cellulosic materials. It found in lower concentration compared to Ascomycota species as recorded by Krakova et al. (2018) who stated that few Basidiomycota taxa were revealed on archival items. It is might due to the activation of the germination of basidiospores requires specific conditions, which make it difficult to obtain cultures of characteristic taxa as explained by Nitiu et al. (2019) who found that only *Rhodotorula* genera representative of the phylum Basidiomycota From Egyptian mummies in the museum of natural sciences of La Plata, Argentina.

### Molecular identification of the isolated deteriorated fungal species

**Fig. 2:**
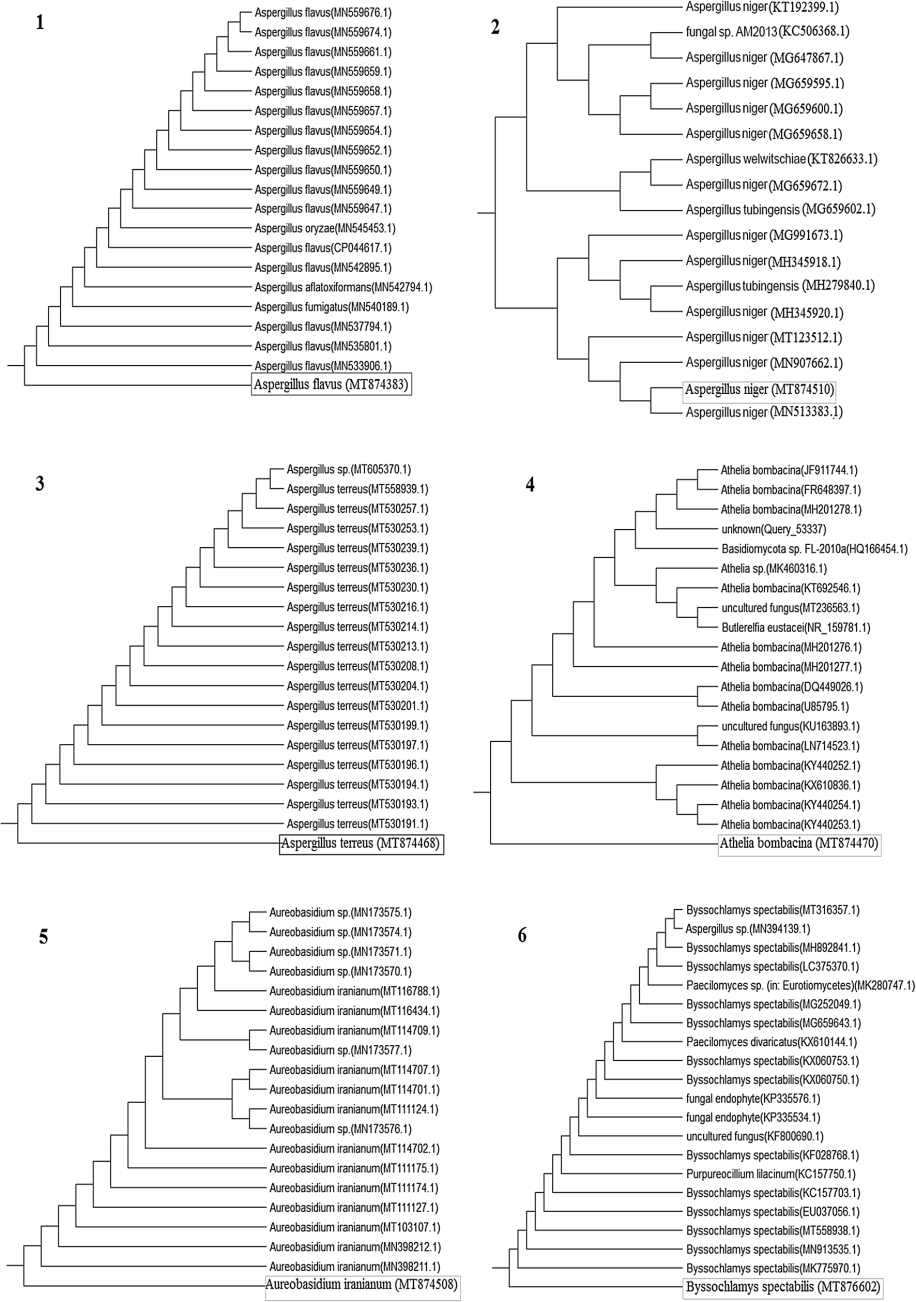
Phylogenetic analysis of the fungal species isolated from the different deteriorated parts of the tested ancient Egyptian mummy cartonnage belonged to the Supreme Council of Antiquities (SCA) excavation in El-Lahun regions, Fayoum, Egypt. (1) *Aspergillus flavus* (MT874383), (2) *A. niger* (MT874510), (3) *A. terreus* (MT874468), (4) *Athelia bombacina* (MT874470), (5) *Aureobasidium iranianum* (MT874508), and (6) *Byssochlamys spectabilis* (MT876602)

**Fig. 3:**
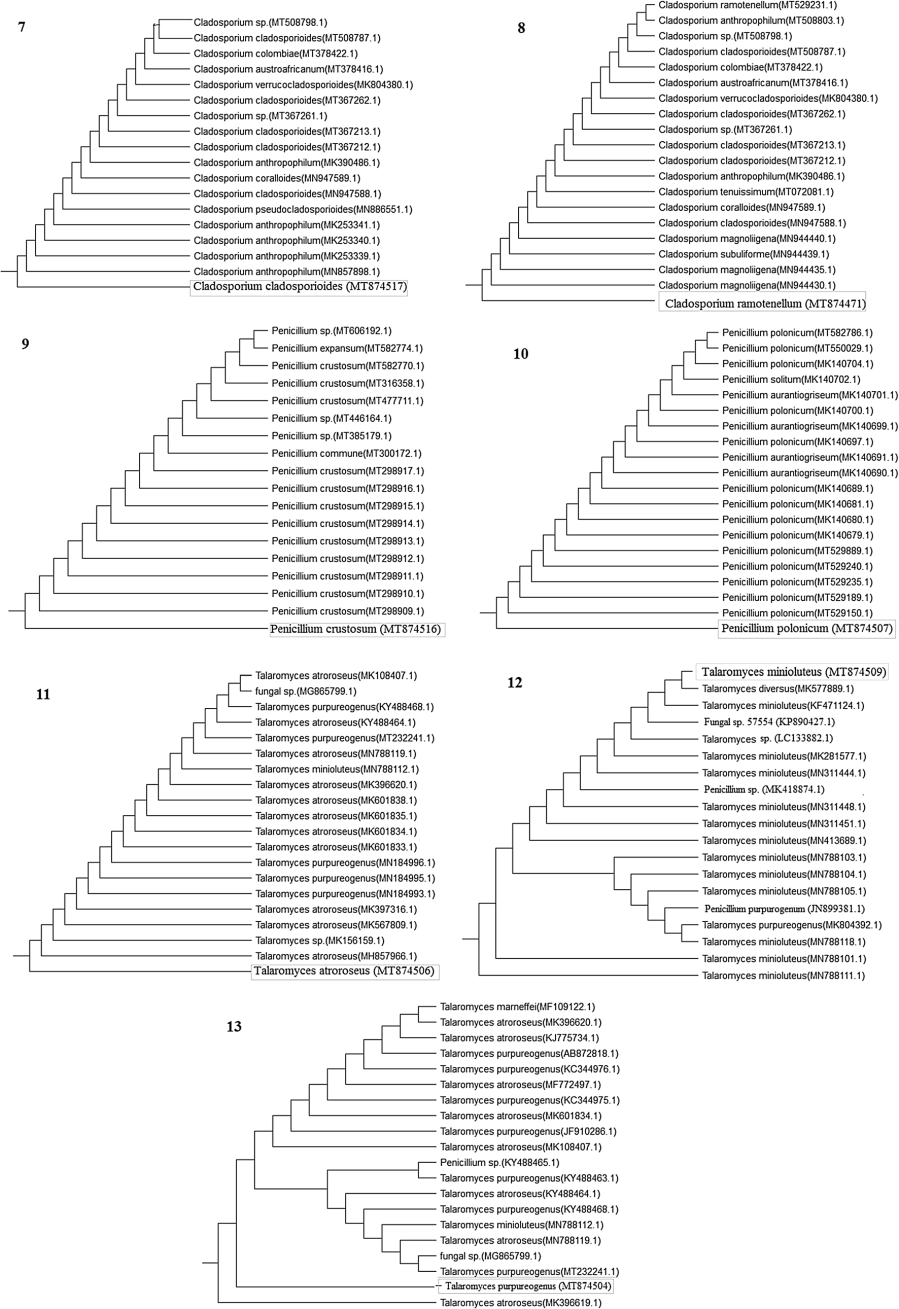
Phylogenetic analysis of the fungal species isolated from the different deteriorated parts of the tested ancient Egyptian mummy cartonnage belonged to the Supreme Council of Antiquities (SCA) excavation in El-Lahun regions, Fayoum, Egypt. (7) *Cladosporium cladosporioides* (MT874517), (8) *C. ramotenellum* (MT874471), (9) *Penicillium crustosum* (MT874516), (10) *P. polonicum* (MT874507), (11) *Talaromyces atroroseus* (MT874506), (12) *T. minioluteus* (MT874509), and (13) *T. purpureogenus* (MT874504)

In the present study, the isolated deteriorated fungal species from the tested ancient Egyptian mummy cartonnage have been determined by PCR amplification and sequencing as follows: *Aspergillus flavus* (MT874383), *A. niger* (MT874510), *A. terreus* (MT874468), *Athelia bombacina* (MT874470), *Aureobasidium iranianum* (MT874508), *Byssochlamys spectabilis* (MT876602), *Cladosporium cladosporioides* (MT874517), *C. ramotenellum* (MT874471), *Penicillium crustosum* (MT874516), *P. polonicum* (MT874507), *Talaromyces atroroseus* (MT874506), *T. minioluteus* (MT874509), and *T. purpureogenus* (MT874504) (Table 2 and Fig. 2 and 3). The most dominant isolated fungal species from the tested ancient Egyptian mummy are belonging to Ascomycota and Basidiomycota phylum. Šimonovičová et al. (2015) recorded Ascomycota, Zygomycota, and Basidiomycota on different materials of mummified human remains (funeral clothes, skin, muscles, and bones) in the Kuffner family crypt in Sládkovičovo, Slovakia. The obtained results agree with Abdel-Maksoud and El-Amin (2013) who reported that the most dominant isolated fungal species from the mummy from the Late Period in ancient Egypt are belonging to Ascomycotina and Zygomycotina genera. The percentage (%) of fungi were: *Pencillium egyptiacum* (25%), *Aspergillus fumigates* (18.75%), *Aspergillus niger* (12.5%), *Pencillium chrysogenum* (12.5%), *Rhizopus arrhizus* (12.5%) and *Rhizopus nigricans* (12.5%), and *Aspergillus flavus* (6.25%).

**Table 2:** Molecular identification of the fungal species isolated from the different deteriorated parts of the tested ancient Egyptian mummy cartonnage belonged to the Supreme Council of Antiquities (SCA) excavation in El-Lahun regions, Fayoum, Egypt

Sample ID	Closest related strain	Genebank Accession number	Similarity (%)	Phylum
1	*Aspergillus flavus*	MT874383	100	Ascomycota
2	*A. niger*	MT874510	100	Ascomycota
3	*A. terreus*	MT874468	100	Ascomycota
4	*Athelia bombacina*	MT874470	99.81	Basidiomycota
5	*Aureobasidium iranianum*	MT874508	99.65	Ascomycota
6	*Byssochlamys spectabilis*	MT876602	100	Ascomycota
7	*Cladosporium cladosporioides*	MT874517	100	Ascomycota
8	*C. ramotenellum*	MT874471	100	Ascomycota
9	*Penicillium crustosum*	MT874516	100	Ascomycota
10	*P. polonicum*	MT874507	100	Ascomycota
11	*Talaromyces atroroseus*	MT874506	100	Ascomycota
12	*T. minioluteus*	MT874509	99.36	Ascomycota
13	*T. purpureogenus*	MT874504	100	Ascomycota

### Synthesis of chalcone derivatives

**Scheme 1:**
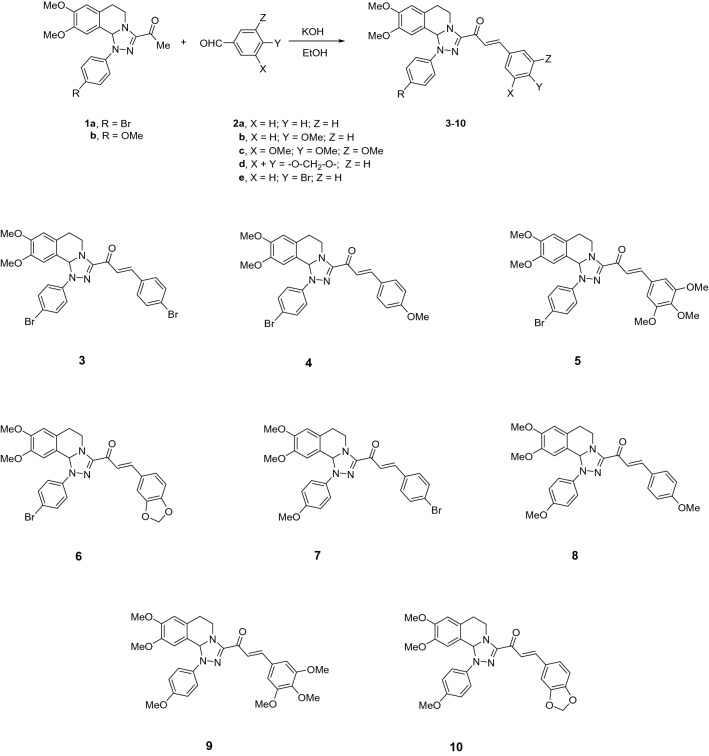
Synthesis of novel tetrahydro-[1,2,4]triazolo[3,4-a]isoquinoline chalcones **3–10**

The starting 3-acetyl-8,9-dimethoxy-1-phenyl-1,5,6,10b-tetrahydro-[1,2,4]triazolo[3,4-*a*]isoquinoline **1** was obtained following the reported procedures via the reactions of 3,4-dihydro-6,7-dimethoxyisoquinoline with nitrilimines (Elwan et al. 1996; Hassaneen et al. 2011). Claisen–Schmidt condensation of compound acetyl compound **1** with the mole equivalents of substituted aldehydes **2** in the presence of potassium hydroxide solution at room temperature affords of the corresponding chalcone derivatives **3–10** (Scheme 1). The constitution of the formed chalcones was proved by careful inspection of the different spectral tools. ^1^H NMR spectra of chalcone derivative number **6** as a representative example indicated two characteristic singlet signals at 3.48 and 3.72 ppm for two methoxy groups. The isoquinoline-H5 and H6 groups appeared as multiplets at 2.64, 3.71, and 4.23 ppm. The –OCH_2_O– group appeared as a singlet at 6.01 ppm. The isoquinoline H10b, H7, and H10 appeared as three singlets at 6.01, 6.61, and 6.87 ppm. Besides, it revealed the vinyl and aromatic protons as multiplets at δ 6.96–7.56 ppm. Moreover, the ^13^C NMR spectra of compound **6** indicated 26 different types of carbon signals appeared at their expected positions.

### Effect of the new eight tested chalcone derivatives on the growth of the isolated deteriorated fungal species from the tested ancient Egyptian mummy cartonnage

The tested eight chalcone derivatives at a concentration of 1 mg/mL showed antifungal activities against thirteen fungal species that were collected from the tested ancient Egyptian mummy cartonnage as shown in Table 3. The predominant majority of the new tested chalcone derivatives provided effective growth inhibition on the tested isolated fungal species which may be due to the presence of nitrogen atoms as recorded by Srilaxmi et al. (2021) who stated that hetero-aromatic skeletons consist of nitrogen atoms are very significant intermediates because of their numerous biological applications. Wang et al. (2010) showed that the synergistic effect of biological activity depends on the combination of chalcone and 1,2,3-triazole are conjugated. Prasath et al. (2015) synthesized quinolinyl chalcones having a pyrazole ring, which was screened for antibacterial and anti-fungal activity against various gram-positive and gram-negative bacteria, and fungal strains. The compounds proved to be most potent with 20.2 mm against Candida. albicans and 21.4 mm activity against Escherichia coli, respectively, where the presence of electron-withdrawing groups improved the antimicrobial activities. The presence of reactive α, β-unsaturated enone group (_CO–CH=CH–) in chalcones derivatives enable them to exhibit a wide range of biological activities (ur Rashid et al. 2019). Chalcones are well-known chemical groups associated with several biological activities such as antibiotic, anti-inflammatory, and antitumor activities (Silva et al. 2022).

**Table 3:** Antifungal activities represented by inhibition percent (%) of the eight tested chalcone derivatives on the thirteen isolated deteriorated fungal species from the tested ancient Egyptian mummy cartonnage at concentration 1 mg/mL

Fungal species	Chalcone derivatives									LSD at 5%
	3	4	5	6	7	8	9	10	Itraconazole	
*Aspergillus flavus*	28.6^Ab^ ± 1.6	41.0^Bcde^ ± 1.9	52.4^Dbc^ ± 2.5	49.5^CDef^ ± 1.9	27.6^Acd^ ± 1.0	45.7^BCd^ ± 1.6	64.8^Ed^ ± 0.9	51.4^Dde^ ± 1.6	25.7^Aa^ ± 1.6	5.18
*A. niger*	29.7^Db^ ± 0.7	8.0^Ba^ ± 1.5	69.5^Fe^ ± 2.2	0.0^Aa^ ± 0.0	33.3^Dde^ ± 0.7	49.3^Ed^ ± 1.9	76.8^Ge^ ± 2.6	16.7^Ca^ ± 0.7	66.7^Fg^ ± 0.7	10.73
*A. terreus*	48.9^Cde^ ± 1.1	54.4^Cf^ ± 2.9	62.2^Dde^ ± 2.9	25.6^Bbc^ ± 2.9	51.1^Cf^ ± 2.9	12.2^Aa^ ± 2.9	76.7^Ee^ ± 0.0	26.7^Bb^ ± 1.9	47.8^Ccde^ ± 1.1	7.77
*Athelia bombacina*	52.4^Ce^ ± 2.4	35.7^Bcd^ ± 4.1	97.6^Ef^ ± 2.4	52.4^Cefg^ ± 2.4	21.4^Abc^ ± 0.0	47.6^Cd^ ± 2.4	100.0^Eg^ ± 0.0	45.3^Cd^ ± 2.4	66.7^Dg^ ± 2.4	10.14
*Aureobasidium iranianum*	30.0^Bb^ ± 0.0	16.7^Ab^ ± 3.3	36.7^Ba^ ± 3.3	30.0^Bcd^ ± 0.0	36.7^Be^ ± 3.3	20.0^Aabc^ ± 0.0	96.7^Dg^ ± 3.3	33.3^Bbc^ ± 3.3	50.0^Cde^ ± 0.0	9.13
*Byssochlamys spectabilis*	45.9^Fcd^ ± 2.7	0.0^Aa^ ± 0.0	31.1^Da^ ± 1.3	21.5^Bb^ ± 0.7	25.9^Cc^ ± 2.0	64.4^Ge^ ± 1.3	84.4^If^ ± 1.3	35.6^Ec^ ± 1.3	78.5^Hh^ ± 0.7	10.65
*Cladosporium cladosporioides*	48.5^Bde^ ± 3.0	42.5^Bde^ ± 3.0	100.0^Df^ ± 3.0	57.5^Cg^ ± 3.0	39.4^Be^ ± 3.0	15.1^Aab^ ± 3.0	100.0^Dg^ ± 0.0	45.5^Bd^ ± 5.2	42.5^Bbc^ ± 3.0	10.84
*C. ramotenellum*	42.9^Acd^ ± 2.0	46.4^Aef^ ± 2.0	66.7^Dde^ ± 1.2	55.9^Bfg^ ± 1.2	53.6^Bf^ ± 2.0	69.1^De^ ± 1.2	100.0^Eg^ ± 0.0	69.1^Df^ ± 1.2	60.7^Cf^ ± 0.0	6.48
*Penicillium crustosum*	52.8^DEe^ ± 2.8	33.3^ABc^ ± 4.8	58.3^Ecd^ ± 4.8	36.0^BCd^ ± 2.7	38.9^BCe^ ± 2.8	25.0^Ac^ ± 0.0	38.9^BCa^ ± 2.8	51.5^DEde^ ± 1.5	44.5^CDbcd^ ± 2.8	4.40
*P. polonicum*	45.0^Ccd^ ± 3.3	45.0^Ce^ ± 3.3	93.5^Df^ ± 3.2	32.0^Bcd^ ± 0.0	15.8^Ab^ ± 3.2	22.3^Abc^ ± 5.6	48.2^Cb^ ± 3.3	54.7^Ce^ ± 3.2	51.5^Ce^ ± 0.0	8.74
*Talaromyces atroroseus*	0.0^Aa^ ± 0.0	0.0^Aa^ ± 0.0	49.1^Fb^ ± 3.5	0.0^Aa^ ± 0.0	7.0^Ba^ ± 1.7	19.3^Cabc^ ± 1.8	100.0^Gg^ ± 0.0	29.8^Dbc^ ± 3.5	40.3^Eb^ ± 1.8	12.54
*T. minioluteus *	41.7^Ec^ ± 1.7	23.3^Cb^ ± 1.7	58.3^Gcd^ ± 1.7	5.0^Aa^ ± 0.0	25.0^Cc^ ± 2.9	15.0^Bab^ ± 2.9	71.7^He^ ± 1.7	35.0^Dbc^ ± 2.9	50.0^Fde^ ± 2.9	8.27
*T. purpureogenus*	0.0^Aa^ ± 0.0	35.9^Ccd^ ± 2.6	61.5^Ede^ ± 0.0	48.7^De^ ± 5.1	25.7^Bc^ ± 2.6	43.6^CDd^ ± 2.6	58.9^Ec^ ± 2.6	35.9^Cc^ ± 2.6	41.1^CDb^ ± 2.6	7.2
LSD at 5%	5.78	5.92	6.98	6.68	4.35	6.35	6.76	4.59	4.51	

Numbers expressed as mean ± standard error (*n* = 3) for each sample. Different capital letters in the same raw show mean values of at a significant level (*p* < 0.05), while different small letters in the same column show mean values of at a significant level (*p* < 0.05)

### Structural activity relationship (SAR)


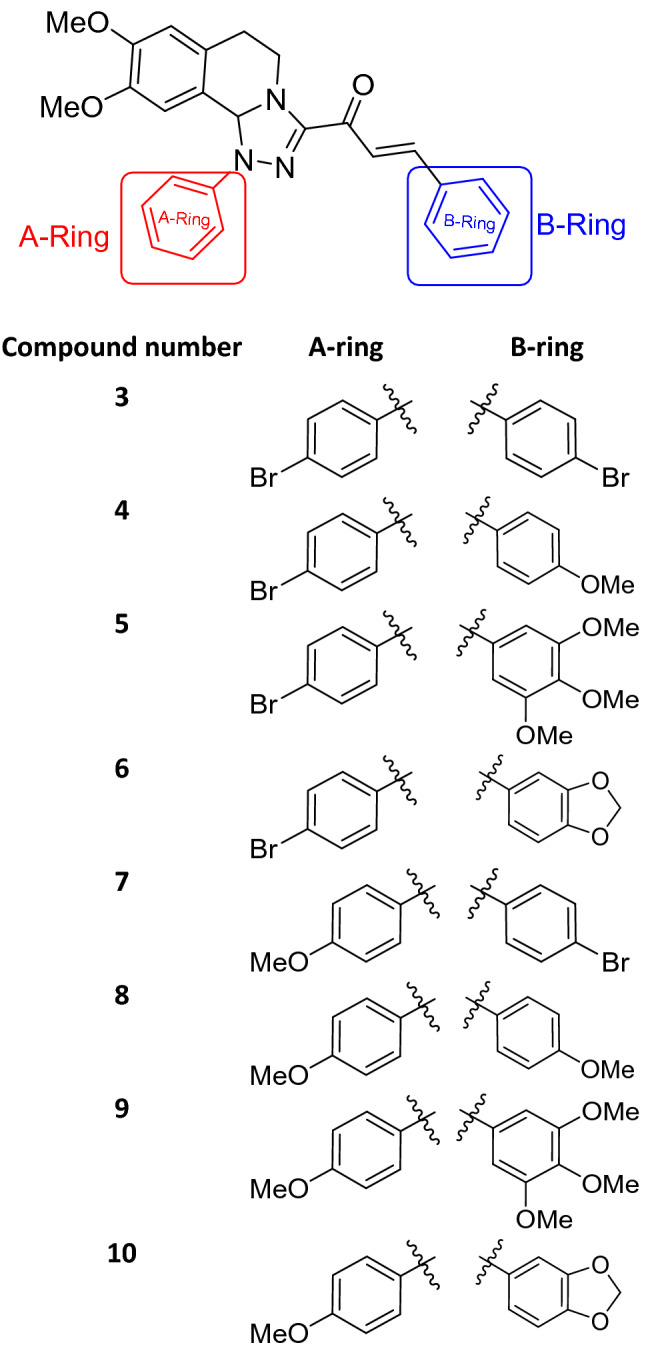


The structure can be seen as *α,β*-unsaturated enone group attached to two rings (A-ring and B-ring). A-ring represents the acetyl part of chalcone, which, is the aryl group attached to [1,2,4]triazolo[3,4-*a*]isoquinoline group. B-ring represents the aldehyde part. All compounds have two fixed methoxy groups in the base structure. The additional electron-donating groups such as methoxy groups increase the activity as shown in Table 1.

The new tested chalcone derivative **9** is the best inhibitor for the isolated deteriorated fungal species, which has the highest significant inhibition (100%) on the growth of *Athelia bombacina, Cladosporium cladosporioides, C. ramotenellum* and *Talaromyces atroroseus*. The activity of the chalcone compound may be due to the presence of six methoxy groups (three methoxy group in A-ring and three methoxy groups in B-ring). The results were found to be in accordance with that reported by Verma et al. (2020) who stated that the compounds bearing electron-donating group such as methyl on phenyl ring exhibited better activity against fungal strains. Also, the antimicrobial activity may refer to the less bulk compound as recorded by Geweely (2009b) who stated that the new complex of a tetrahedral geometry (less bulk) that enhances the rate of entrance through the candidal cell and accordingly increase its anticandidal activity and it can be used in future as novel, more active drug for designing new chemotherapeutic agents. Mohamed et al. (2012) suggested that chalcone derivatives containing electron-releasing groups as OCH_3_ increase the antimicrobial activity.

The 2′,4′-dihydroxy-3′-methoxychalcone and the 2′,4′-dihydroxychalcone applied on *Alternaria alternate, Colletotrichum truncatum*, *Fusarium equiseti*, *F. graminearum, F. verticillioides, Phomopsis longicolla* and *Sclerotium bataticola*, which completely suppressed the growth of *C. truncatum* and *P. longicolla* at the concentration of 6.25 µg/mL (Jimenez et al. 2014). Also, Jayasinghe et al. (2004) found that five chalcones isolated from *Artocarpus nobilis* have antifungal efficiency against *Cladosporium cladosporioides*, and *Aspergillus niger*.

The new chalcone derivative **5** and **7** came next in rank of antifungal activity and causes complete significant inhibition (100%) of *Cladosporium cladosporioides* followed by *Athelia bombacina* (97.6%) and *P. polonicum* (93.5%), while the least percent of inhibition was shown on *Byssochlamys spectabilis* (31.1%). It has one two methoxy and one bromo group in A-ring and three methoxy groups in B-ring. The presence of halogen substituent (Br) causes more bulk compound which may also act as electron-withdrawing group is responsible for the antimicrobial activity (Rani et al. 2019). The activity of compound **5** is less than activity of compound **9** which may be due to the presence of one bromine group (Br) (as an electron-withdrawing group) instead of methoxy group (OMe) in A-ring. Burmaoglu et al. (2020) reported that many organo-halogen contains bromine have been recorded to have potential antimicrobial and antioxidant efficiency and also inhibited protein tyrosine phosphatase, and aldose reductase activity.

The tested chalone derivatives **8** and **10** presented moderate inhibitory activity against tested isolated fungal species. ElSohly et al. (2001) isolated chalcones from the *Maclura tinctoria*, which showed inhibitory activity against *Candida albicans* and *Cryptococcus neoformans*. Chalcones are multifunctional molecules, which can show multiple biological activities. licochalcone A act as cell cycle inhibitor (Díaz-Tielas et al. 2016). Most chalcones inhibit the biosynthesis of the yeast cell wall and thus unfold their antifungal potential. The effects of several substitutions of synthetic chalcones against *Candida albicans* depended on their ability to interact with sulfhydryl groups. Chalcones have been also tested against *Cryptococcus neoformans*, *Candida*, *Microsporum*, *Trichophyton* and *Penicillium* species (Lahtchev et al. 2008).

The new synthesized chalcone compound **4** have no activity against *Byssochlamys spectabilis* and *Talaromyces atroroseus,* which may be due to the presence of bromine substituent (Br) as an electron-withdrawing group (Br) and methoxy group (OMe) as an electron-donating group, while chalcone derivative **6** have no activity against *A. niger,* and *Talaromyces atroroseus.* Also, new chalcone derivative **3** has no activity against *Talaromyces atroroseus* and *T. purpureogenus*. The antifungal activity of chalcones has been largely attributed to the reactive enone moiety. The enone unit binds to thiol groups of certain proteins in microbial species. The reactions of chalcones are facilitated by electron-withdrawing (EW) groups at p-position for the thiol attack, which showed antifungal properties (Lahtchev et al. 2008). The presence of an enone linkage would be a structural requirement necessary but not by itself sufficient for the antifungal activity (Nowakowska 2007). López et al. (2001) reported that electron-withdrawing groups in the para-position increased the potency of chalcone, while the presence of these groups in the ortho-position of the ring could introduce important effects that result from the size of substituents and the repulsion between them.

### Determination of minimum inhibitory concentration (MIC) for the isolated deteriorated fungal species

The MIC of the eight tested chalcone derivatives on the thirteen isolated deteriorated fungal species from the tested ancient Egyptian mummy cartonnage, El-Lahun regions, Fayoum government, Egypt are presented in Table 4. The MIC of the most efficient chalcone derivatives **13** was ranging from 1 to 3 mg/mL for all isolated deteriorated fungal species. *Athelia bombacina, Cladosporium cladosporioides, C. ramotenellum,* and *Talaromyces atroroseus* were the most susceptible species to the lowest concentration of chalcone derivative number **9** (1 mg/mL). Chalcone derivatives with heterocycle moiety (azoles) are another effective approach to improve antifungal activity. Chalcone derivatives, in which quinolinyl N-oxide was used as a B-ring in chalcones, showed antifungal activities against *C. gattii* (MIC = 7.80 μg/mL) and *Paracoccidioides brasiliensis* (MIC = 1.90 μg/mL) (de Carvalho Tavares et al. 2011). A chalcone derived from carbazoles was assayed for antibacterial activity against two gram-positive (*S. aureus* and *B. subtilis*) and two gram-negative bacterial strains *(E. coli* and *K. pneumonia)* and antifungal activity against *Fusarium oxysporum, Aspergillus niger,* and *Aspergillus flavus* (Ashok et al. 2016). Konidala et al. (2021) found that the antimicrobial screening of the chalcone compounds showed potent antifungal activity with MIC value of 10 µM and 11 µM against *Aspergillus niger*.

**Table 4:** Minimum inhibitory concentrations (MIC) of the eight tested chalcone derivatives on the thirteen isolated deteriorated fungal species from the tested ancient Egyptian mummy cartonnage belonged to the Supreme Council of Antiquities (SCA) excavation in El-Lahun regions, Fayoum, Egypt

Fungal species	Chalcone derivatives (mg/mL)								Itraconazole (µg/mL)
	7	8	9	10	11	12	13	14	
*Aspergillus flavus*	5	5	4	4	5	5	3	5	4
*A. niger*	4	6	3	6	5	3	3	6	2
*A. terreus*	5	4	3	5	4	5	2	5	2
*Athelia bombacina*	3	4	2	3	5	3	1	3	1
*Aureobasidium iranianum*	4	5	3	4	3	2	2	4	1
*Byssochlamys spectabilis*	4	5	4	6	4	3	2	5	1
*Cladosporium cladosporioides*	4	4	1	3	4	5	1	3	1
*C. ramotenellum*	4	4	3	3	3	2	1	2	2
*Penicillium crustosum*	3	4	2	3	3	3	3	2	1
*P. polonicum*	4	4	2	4	5	3	2	3	1
*Talaromyces atroroseus*	6	5	3	5	4	3	1	4	2
*T. minioluteus*	4	5	3	5	4	5	2	5	2
*T. purpureogenus*	5	3	2	3	3	2	2	3	2

Azole and non-azole chalcones derivatives showed effective antifungal activity against *C. albicans* due to inhibit ergosterol biosynthesis and down-regulated ERG11(Sterol 14-demethylase) gene expression (Ahmad et al. 2017). Pyrazine analogs of chalcones showed antifungal activities against *Candida spp.* (MIC = 7.81 µg/ mL), *Aspergillus fumigatus* (MIC = 31.25 µg/mL), and *Trichophyton mentagrophytes* (MIC = 31.25 µg/mL) (Kucerova-Chlupacova et al. 2016). Imidazole–chalcone derivative, exerted effective activities against *Candida* strains with MIC values ranging from 0.78 to 3.125 µg/mL and significantly decreased ergosterol level of *C. krusei* (Osmaniye et al. 2018). The series of 1,4-disubstituted-1,2,3- triazole derivatives of chalcones showed potent activities against *Candida strains* (MIC = 6.5–12.5 µg/mL), *C. neoformans* (MIC = 12.5 µg/mL), *Aspergillus fumigatus* and *A. niger* (MIC = 12.5 µg/mL) (Kant et al. 2016). The high oxidizing power and spontaneous decomposition also make safety, as a common sanitizing agent (Geweely 2011). Some Dehydroacetic acid-chalcone-triazole hybrids were synthesized and evaluated for their antifungal effects by Lal et al. (2018).

The structure–activity relationship showed that the presence of electron-donating groups (OCH_3_ and CH_3_) on the phenyl ring highly enhances the antimicrobial properties (Xu et al. 2019). Mohamed et al. (2012) found that chalcone, compounds containing electron-releasing groups such as OCH_3_ increase the antibacterial activity against *Staphylococcus aureus, Streptococcus faecalis, Bacillus subtilis, Escherichia coli, Pseudomonas aeuroginosa, Neisseria gonorrhoeae* with MIC 20 mg/mL. Andrade et al. (2018) showed that chalcones have antifungal activity against *Trichophyton rubrum* with a MIC of 7.5 μg/mL, while 2′4′-dihydroxychalcone inhibited the growth of *Candida spp.* at a concentration of 15.6 μg/mL, and the action of chalcone was involved in the ergosterol and fungal membrane.

The growth of *Cladosporium cladosporioides* was found more susceptible to the new tested chalcone derivative **5** when exposed to the lowest concentration (1 mg/mL). The tested chalcone derivative **5** at 4 mg/mL showed complete significant inhibition against *Aspergillus flavus,* and *Byssochlamys spectabilis.* The MIC of the chalcone derivative **5** was ranging from 1 to 4 mg/mL for all isolated deteriorated fungal species. The structure–activity relationship revealed that the position of the bromo atom on the benzene ring is highly favored for antimicrobial activity. This may be attributed to the greater hydrophobic effect of bromo group at 4-position (Abdullah et al. 2014). Chalcone derivatives with electron-withdrawing substituents (-Br) at 2 and 4-position on phenyl ring showed increases in the cytotoxicity level of tested compounds (Xu et al. 2019). The introduction of different electronic properties of two functional groups (–Br and –OMe) on phenyl rings exhibited superior antimicrobial activity (Yadav et al. 2017).

The MIC of the chalcone derivative **8** was ranging from 2 to 5 mg/mL for all isolated deteriorated fungal species. *Aspergillus flavus, A. terreus, Cladosporium cladosporioides,* and *T. minioluteus* were completely inhibited at 5 mg/mL of chalcone derivative **8***.* Dimeric chalcone isolated from *Mallotus philippinensis* exhibited inhibitory activity that inhibits 50% of the microbial growth of (MIC50) are 4–8 and 16 μg/mL against *Cryptococcus neoformans* and *Aspergillus fumigatus*, respectively (Kulkarni et al. 2014). Prenylated chalcone isolated from the *Maclura tinctoria* leaves showed antifungal activities against *Candida albicans* and *C. neoformans* (MIC50 = 15 μg/mL and 7 μg/mL, respectively) (ElSohly et al. 2001). Licochalcone-A showed antifungal activity against *C. albicans* and *Trichophyton rubrum* with minimum inhibitory concentration (MIC) 62.5–150 μM and 11.52 μM, respectively by induction of genes related to the ergosterol biosynthesis pathway and genes encoding enzymes involved in cell-wall synthesis and glyoxylate cycle (Seleem et al. 2016).

The growth of four fungal species (*Aureobasidium iranianum, C. ramotenellum, Penicillium crustosum,* and *T. purpureogenus*) were the most sensitive species to new chalcone derivative **7** with percent of inhibition at 100% at 3 mg/mL, while *Aspergillus flavus, A. niger, Athelia bombacina,* and *P. polonicum* were completely inhibited with 5 mg/mL. The MIC of the chalcone derivative **7** was ranging from 3 to 5 mg/mL for all isolated deteriorated fungal species. An unsubstituted chalcone showed antifungal activity against *Trichophyton rubrum* with a MIC of 7.5 μg/mL and it reduced ergosterol content, while the enzymatic activity of Fas Cell Surface Death Receptor (FAS gene) was inhibited with values of 68.23 and 17.1 μg/mL (Bitencourt et al. 2013). 2′4′-dihydroxychalcone at a concentration of 15.6 μg/mL inhibited the growth of *Candida spp.* by involving in the ergosterol and fungal membrane (Andrade et al. 2018). Jin (2019) showed that the chalcone skeleton was also found to be fundamental for antifungal activity. Lahtchev et al. (2008) demonstrated that chalcones may react with some proteins involved in cell separation and DNA was not the main target. Burmaoglu et al. (2017) reported that the fluoro atoms in the ring of 2′, 4′, 6′-trimethoxychalcone did not enhance antifungal activity. Diazenyl chalcones showed antifungal activity against *Aspergillus fumigatus* (MIC = 31.25 μg/mL) and *C. albicans* (MIC = 15 μg/mL) (Kaur and Narasimhan 2018).

The MIC of the chalcone derivative **10** was ranging from 2 to 6 mg/mL for all isolated deteriorated fungal species. *C. ramotenellum,* and *Penicillium crustosum* were completely inhibited with 2 mg/mL of chalcone derivative **10**. Nowakowska (2007) showed that the most active chalcone compound does not have an electron-withdrawing group in the para-position of ring A, but also does not have a substituent in ortho-position. This compound showed strong antifungal activities against *Microsporum canis* (MIC ¼ 25 mg/mL), *Microsporum gypseum* (1.5 mg/mL), *Trichophyton mentagrophytes* (3 mg/mL), *Trichophyton rubrum* (3 mg/mL) and *Epidermophyton floccosum* (0.5 mg/mL).

The least and last antifungal activities were shown with the new chalcone derivatives **3**, **4**, and **6**, where the MIC of three chalcone derivatives was ranging from 3 to 6 mg/mL. *A. niger* require a higher concentration (6 mg/mL) of chalcone derivatives **4**, and **6** for complete inhibition. Kucerova-Chlupacova et al. (2016) reported that 2-bromo or 4-bromo substitution had an inhibiting effect on the growth of *T. interdigitale* (MIC 3.9–7.81 µmol/L), while halogenated chalcone derivatives also inhibited the growth of *Candida* spp. Wei et al. (2016) examined chalcone derivatives against Gram-positive (*Staphylococcus aureus* and *Streptococcus mutans*), four Gram-negative (*Escherichia coli*, *Salmonella typhimurium,* and *Pseudomonas aeruginosa*) and fungus (*Candida albicans*) strain with MIC ranging from 1 to 64 µg/mL. Muškinja et al. (2016) have reported a series of ferrocenyl chalcones showed antibacterial and antifungal activity against five species of bacteria (*Staphylococcus aureus, Bacillus cereus, B. subtilis, Escherichia coli, and Proteus mirabilis*) and five species of fungi (*Aspergillus niger, Candida albicans, Penicillium italicum, Mucor mucedo, Trichoderma viride*). The measured MIC values of synthesized compounds were in the range of 0.0352–0.8873 mg/mL. Xu et al. (2019) suggested that chalcone derivatives were a promising candidate for the development of new antimicrobial agents in the future.

## Conclusion

The essential and crucial component of both the Egyptian and the world's cultural legacy are mummies. Its susceptibility to infection by fungal colonies influenced by the range of materials utilized and environmental variables. The impact of microbial activity on the deterioration of mummy cartonnage is a global problem along with its disinfection over time is challenging for preserving them for the next generation. The Ascomycota (*Aspergillus flavus*, Aspergillus *terreus* and by *A. niger)* made up majority of the isolated core phyla. Application of the most efficient new chalcone derivative **9** {(*E*)-1-(8,9-Dimethoxy-1-(4-methoxyphenyl)-1,5,6,10b-tetrahydro-[1,2,4]triazolo[3,4-*a*]isoquinolin-3-yl)-3-(3,4,5 trimethoxyphenyl)prop-2-en-1-one} with three methoxy groups as an electron-donating group and one methoxy group (electron-withdrawing group) can be successfully used with minimum inhibitory concentration (MIC) ranging from 1 to 3 mg/mL against fungal deterioration instead of using physical and chemical disinfection to avoid the unfavorable effects on the artifacts, public health and environment.

## Data Availability

Data available at request.
